# Long-term immune checkpoint inhibition therapy for advanced stage Merkel cell carcinoma

**DOI:** 10.1016/j.jdcr.2025.10.027

**Published:** 2025-10-27

**Authors:** Marisa R. Diiorio, Nicole Trepanowski, Payal Shah, Keisuke Shirai

**Affiliations:** a Geisel School of Medicine at Dartmouth, Hanover, New Hampshire; b Department of Dermatology, Dartmouth-Hitchcock Medical Center, Lebanon, New Hampshire; c Hematology/Oncology, Norris Cotton Cancer Center, Dartmouth-Hitchcock Medical Center, Lebanon, New Hampshire

**Keywords:** circulating tumor DNA, immune checkpoint inhibition, Merkel cell carcinoma

## Introduction

Advanced Merkel cell carcinoma (MCC) has a poor prognosis, with a 5-year overall survival rate of approximately 14% for patients with distant metastatic disease, and high recurrence rates of approximately 58% within the first year.^1^^,^^2^ Outcomes for advanced MCC have recently improved with the shift from chemotherapy to immune checkpoint inhibitors (ICIs) targeting programmed cell death protein 1 (PD-1)/programmed cell death ligand 1 (PD-L1) pathways. Studies show that ICI therapy has increased the median progression-free survival in advanced cases to 16.8 months compared to 3 months with traditional chemotherapy (platinum plus etoposide).^2^^,^^3^ Clinical trials for ICI use in advanced MCC continue therapy until a complete response (CR), progression of disease, severe adverse events (AEs), or for up to 2 years.^4^^,^^5^ However, 1 study with a median ICI treatment of 13.5 months did not yield a durable response at 1 year post cessation, whereas another study demonstrated that 39% of patients who discontinued ICI had progression of disease compared to only 14% of patients who continued ICI for a full 2 years, suggesting that patients may benefit from longer and uninterrupted durations of ICI treatment.^6^^,^^7^ However, long-term data for ICI therapy remain limited.^2^ Here, we report 2 cases of advanced multirelapse MCC achieving durable radiographic CR with long-term ICI therapy.

## Case report

### Patient 1

**Fig 1:**
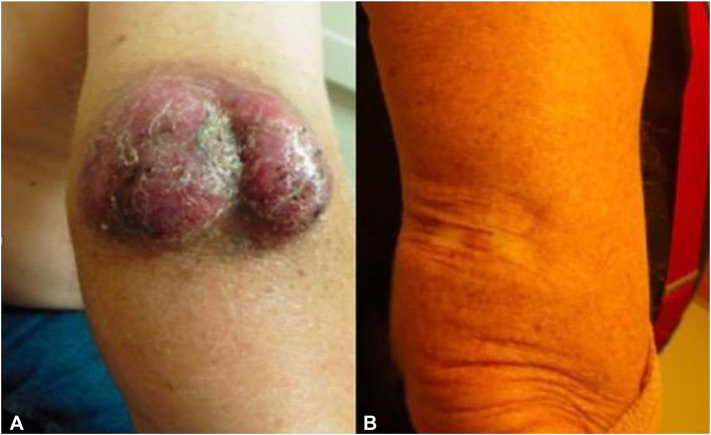
Patient 1 presentation **(A)** before treatment and **(B)** after treatment with avelumab and radiation.

A 62-year-old male patient presented with a tumor on the right arm (Fig 1, *A*) and ipsilateral axillary lymphadenopathy. Skin and nodal biopsy were notable for sheets of small blue cells positive for synaptophysin, CD56, CK20, and neurofilament and negative for CK7 and TTF1. Imaging showed no distant metastases, confirming stage IIIB MCC. Neoadjuvant avelumab (anti-PD-L1) dosed at 1000 mg every 2 weeks, and radiation to the primary tumor and lymph node basin, were started. By cycle 13, he had achieved CR, and surgery was deferred (Fig 1, *B*).

Avelumab was decreased to every 3 weeks at cycle 27 for grade 2 myalgias and ultimately discontinued at cycle 38 given progressively worsening joint pains. Six months later, splenic metastases developed, and rechallenge with pembrolizumab, an anti-PD-1 ICI, dosed at 200 mg every three weeks, was initiated. Rechallenge with avelumab was not performed, given its toxicity profile, including his persistent myalgias, and progression of disease with distant metastasis after cessation. CR was achieved by cycle 6 of pembrolizumab, and treatment was continued due to the high risk of relapse. Dosing was reduced to 200 mg monthly at cycle 16 and then to every other month at cycle 33. In total, he has completed 45 cycles of pembrolizumab (5.3 years) and continues treatment with 200 mg at 3-month intervals with maintained radiographic CR, and circulating tumor DNA (ctDNA) remains undetectable.

### Patient 2

**Fig 2:**
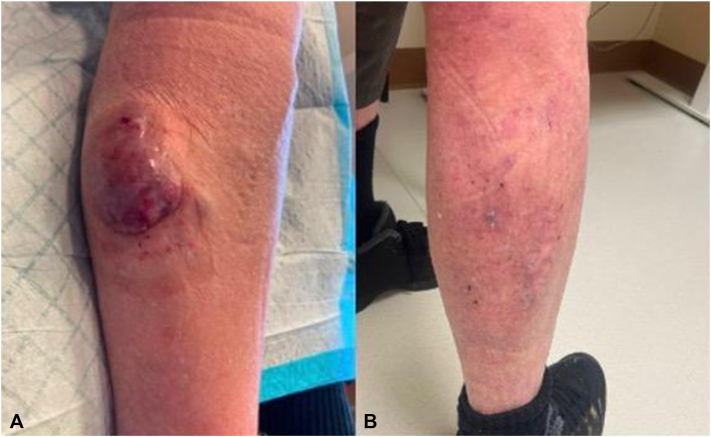
Patient 2 presentation **(A)** before treatment and **(B)** after treatment with pembrolizumab and surgical resection.

A 69-year-old male patient presented with a tumor on the calf of the right leg and swelling on the right leg (Fig 2, *A*). Biopsies of the calf and inguinal lymph node had sheets of small blue cells positive for CK20, chromogranin, and synaptophysin and negative for Merkel cell polyomavirus. Imaging revealed inguinal lymphadenopathy and pelvic metastasis, confirming stage IV MCC. He began 400 mg of pembrolizumab therapy every 6 weeks. After 6 cycles, a near-complete resolution of the calf nodule allowed for successful surgical resection of the primary tumor.

**Fig 3:**
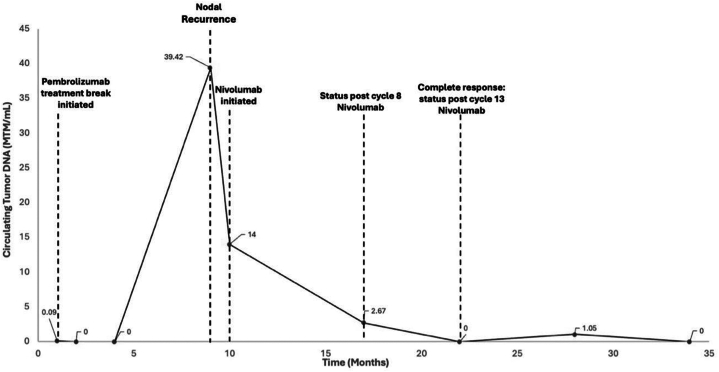
Circulating tumor DNA levels over time for patient 2. *MTM*, Mean tumor molecule.

Adjuvant pembrolizumab was started after local recurrence 2 months later, reaching CR after 4 cycles. Pembrolizumab (400 mg) was discontinued after 13 cycles due to a grade 3 pruritic rash, managed with prednisone and triamcinolone cream. A local recurrence 6 months later prompted resumption of 200 mg every 3 weeks with pembrolizumab therapy. Rechallenge with the same ICI, pembrolizumab, was tried, given his AEs were managed with steroids, and he only had local recurrence. CR was achieved after 4 additional cycles. A 9-month treatment break led to an increase in ctDNA (Fig 3), which prompted further investigation with imaging, and lymph node recurrence was identified.

Despite positive and rapid responses to pembrolizumab, the significant skin toxicity and multiple recurrences during treatment breaks guided the decision to switch to nivolumab, a different anti-PD-1 antibody. Nivolumab (480 mg every 6 weeks) was started with CR by cycle 13 (Fig 2, *B*). The patient has completed 24 cycles of nivolumab (2.3 years) and continues maintenance treatment with undetectable ctDNA levels and no radiographic recurrences. Of note, he has tolerated nivolumab significantly better with minimal skin toxicity.

## Discussion

Here, we report 2 cases of advanced MCC achieving durable radiographic CR after extended ICI therapy (5.3 and 2.3 years, respectively), suggesting prolonged ICI use may be a feasible treatment option for select patients to sustain disease remission. However, identification of patients who may benefit from long-term ICI, as well as the total treatment duration, remains an open question.

Long-term immunotherapy may not be necessary for every patient with advanced MCC, especially when considering high costs and risks of AEs. For most patients, active surveillance with treatment at relapse is appropriate, given that those who recur frequently respond well (50%-70% objective response rates) to rechallenge with the same or an alternative PD-1/PD-L1 inhibitor.^8^ In contrast, patients who progress during or after immunotherapy rechallenge have poor prognoses and very limited treatment options.^6^, ^7^, ^8^ Therefore, as observed in our patient cases, continued maintenance therapy may be appropriate for individuals at high risk of repeat recurrence following ICI rechallenge.

To optimize treatment decisions, better objective markers of relapse risk are needed. More specifically, data indicate that among those who did not complete the full 2 years of treatment, relapse rates are higher for patients who discontinued treatment due to AEs compared to those who stopped electively.^9^ As demonstrated in our cases, switching to an alternative ICI within the same class, rather than discontinuing completely, may mitigate AEs while maintaining therapeutic benefit.

Recent evidence suggests that ctDNA may also serve as a useful biomarker for identifying high-risk patients. A recent prospective multicenter study validated ctDNA as a biomarker for detecting MCC recurrence during surveillance.^10^ Patients with positive ctDNA at any point had significantly higher recurrence rates of MCC than those negative for ctDNA, with higher quantitative ctDNA levels strongly correlating with the increased recurrence risk.^10^ The ctDNA level trends observed in patient 2 highlight the potential value of longitudinal ctDNA monitoring to guide maintenance therapy duration, dosing, and frequency (Fig 3).

These cases suggest that long-term ICI treatment for MCC is feasible, well-tolerated, and can have positive outcomes for patients at high risk of relapse. Larger cohort studies are warranted to more precisely identify high-risk patients and further evaluate the safety, efficacy, and duration of maintenance immunotherapy in this population.

## Conflicts of interest

None disclosed.
